# ‘What men don’t know can hurt women’s health’: a qualitative study of the barriers to and opportunities for men’s involvement in maternal healthcare in Ghana

**DOI:** 10.1186/s12978-015-0083-y

**Published:** 2015-10-10

**Authors:** John Kuumuori Ganle, Isaac Dery

**Affiliations:** Department of Population, Family and Reproductive Health, School of Public Health, University of Ghana, Legon, Ghana; Institute of African and Gender Studies, University of Cape Town, Cape Town, South Africa

**Keywords:** Men, Maternal healthcare, Involvement, Patriarchy, Ghana

## Abstract

**Background:**

The importance of men's involvement in facilitating women’s access to skilled maternal healthcare in patriarchal societies such as Ghana is increasingly being recognised. However, few studies have been conducted to examine men’s involvement in issues of maternal healthcare, the barriers to men’s involvement, and how best to actively involve men. The purpose of this paper is to explore the barriers to and opportunities for men’s involvement in maternal healthcare in the Upper West Region of Ghana.

**Methods:**

Qualitative focus group discussions, in-depth interviews and key informant interviews were conducted with adult men and women aged 20–50 in a total of seven communities in two geographic districts and across urban and rural areas in the Upper West Region of Ghana. Attride-Stirling’s thematic network analysis framework was used to analyse and present the qualitative data.

**Results:**

Findings suggest that although many men recognise the importance of skilled care during pregnancy and childbirth, and the benefits of their involvement, most did not actively involve themselves in issues of maternal healthcare unless complications set in during pregnancy or labour. Less than a quarter of male participants had ever accompanied their wives for antenatal care or postnatal care in a health facility. Four main barriers to men’s involvement were identified: perceptions that pregnancy care is a female role while men are family providers; negative cultural beliefs such as the belief that men who accompany their wives to receive ANC services are being dominated by their wives; health services factors such as unfavourable opening hours of services, poor attitudes of healthcare providers such as maltreatment of women and their spouses and lack of space to accommodate male partners in health facilities; and the high cost associated with accompanying women to seek maternity care. Suggestions for addressing these barriers include community mobilisation programmes to promote greater male involvement, health education, effective leadership, and respectful and patient-centred care training for healthcare providers.

**Conclusions:**

The findings in this paper highlight the need to address the barriers to men’s involvement, engage men and women on issues of maternal health, and improve the healthcare systems – both in terms of facilities and attitudes of health staff - so that couples who wish to be together when accessing care can truly do so.

## Background

In many societies in Africa including Ghana where the burden of maternal mortality is high, it is increasingly being recognised that even though maternal survival requires improvements in comprehensive and basic obstetric care coverage and quality, mobilisation and engagement of men is required to increase the use of skilled delivery services, eliminate delays in accessing care, and promote timely referral when problems arise [1–3]. In patriarchal societies in particular, men are often major decision-makers for the family, hence decisions around when, where, and even if, a woman should have access to healthcare often fall on men [1, 4, 5]. For instance, due to the patriarchal nature of many Ghanaian societies, men often govern behaviours regarding family planning, women's work load, allocation of money, availability of nutritious food, transport and time that women can use to access healthcare services [2, 3]. In the context of maternal health, a study in Tanzania found that households headed by men were associated with more home-based deliveries [6]. In Pakistan, high decision-making power by men was linked to low utilisation of antenatal and delivery care services [7]. A number of recent studies in Ghana have also indicated that men’s disapproval is a major barrier to women’s use of skilled maternal and newborn healthcare services at health facilities [2, 3]. It has also been noted in some parts of the world that husband's approval is an important determinant of access and use of maternal healthcare services [8, 9]. Related studies in the field of HIV/AIDS have similarly underscored the role of men. For example, a study by Mbonye et al. [10] on barriers to contraception among HIV-positive women in a peri-urban district of Uganda found that women’s fear of their spouses' reactions to the test results constrained HIV/AIDS testing.

Due to the roles men play as major decision-makers in facilitating or discouraging access to needed maternal healthcare services, there is growing recognition of the importance of men's involvement in facilitating women’s access to maternal healthcare [1–3,, 11]. Evidence demonstrates that involving men in maternal healthcare has beneficial consequences for access and could significantly influence the health outcomes of women and children [8, 9, 11–17]. Some studies have found that health interventions that target and involve men are strongly associated with enhanced and better birth outcomes and increased use of skilled maternal healthcare services [8, 9, 11–17].

At the same time, the focus of maternal health programming in many African countries including Ghana has primarily been on women, often seeing maternal health issues as women's business [18]. In instances where attempts have been made to involve men in maternal healthcare, such efforts have often been promoted by individual health facilities [1], and as well centred around sexual and reproductive health issues such as condom use, family planning, and prevention of mother-to-child transmission of HIV [19–21]. Consequently, the role and involvement of men as key determinants of access to maternal healthcare has largely been unexplored [22]. There is dearth of evidence on men's views on their involvement in maternal healthcare in Ghana [22]. The purpose of this paper is to explore the barriers to, and opportunities for, men’s involvement in maternal healthcare in Ghana, focusing on the Upper West Region where maternal mortality is quite high. This could deepen understanding of men's involvement in maternal health hence serve as a guide for policy design.

### Conceptual framework

Despite the bourgeoning interest in studying male involvement in maternal healthcare, there is no consensus on how involvement should be conceptualised and what theoretical approaches are relevant to studying male involvement empirically. This notwithstanding, two broad theoretical approaches have been used in the literature [23]. The first approach sees male involvement to be a marker of gender equity as part of the social determinants of health framework [24]. Within this framework, adopting more equitable gender roles such as joint decision-making among couples and shared control of household tasks or parenting is posited to lead to healthier behaviours and improved care-seeking [23]. The second theoretical approach considers male involvement as more instrumental, such that the direct assistance provided by men leads to improvement in their partners’ and children’s health [23]. This approach is therefore seen as ‘gender-neutral’ [25] or ‘genderblind’ [12] in that it considers men’s actions independent of their gendered roles, and may in fact reinforce gender norms that disempower women [26].

In studying the challenges to and opportunities for men’s involvement in maternal healthcare in Ghana, therefore, we believe both of these theoretical approaches are useful. For instance, whereas practical activities such as attending childbirth may be influenced by men’s desire to challenge traditional gender norms and combat gender inequalities that contribute to women’s poor health, an instrumental approach might see such a behaviour (such as attending birth) as an end in itself, that is a way to improve the health of mother and child. In this way whether men believe in gender equality and gender role differentiation, and whether they believe their involvement in maternal healthcare may produce positive health outcomes, could enhance or inhibit their willingness to involve in maternal healthcare. In this sense, both gender norms and instrumental or pragmatic imperatives could dictate whether or not men become involved in supporting their spouses to seek pregnancy and birthing care. Indeed, as our findings will illustrate, men’s involvement or non-involvement in maternal health in the Upper West Region is shaped by both notions of gender roles and more instrumental motivations related to a desire to improve maternal and child health outcomes.

## Methods

### Research design

This paper forms part of a larger study that the authors conducted between January and August 2014 to examine men’s involvement in maternity care in Ghana. The study employed a qualitative research design. A qualitative research approach is useful as it rests on the idea that social reality and experiences are complex and dynamic. It has been argued that the quantitative research method fails to fully comprehend the dynamics of social life and, in particular, ignores local people’s perspectives and understanding [27]. Since this research aimed to explore the barriers to, and opportunities for, men’s involvement in maternal healthcare, the use of a qualitative approach was ideal [28]. The qualitative design provided a detailed description of men’s perceptions, attitudes and involvement in maternal healthcare and uncovered how women navigate their maternal healthcare needs in Ghana's Upper West Region.

### The research context

The study was conducted in seven communities across two districts (Wa West and Lambussie-Karni districts) of the Upper West Region (UWR) of Ghana. The UWR is located on the northwestern part of Ghana, and covers a geographical area of 18,478 square km. The region has a population of 702,110, comprising 51.4 % females and 48.6 % males [29]. The region is the second poorest region in Ghana, with 82.5 % of the total population characterised as rural [19]. The primary economic activity is agriculture, with approximately 72 % of the active labour force engaged in agriculture [29]. Women constitute the majority of the farm labour-force. Women also make up the majority of contributors in the informal sector in the region.

In the UWR, only 12.5 % of roads are tarred, with most in poor and deplorable condition, becoming impassable in the rainy season [30]. In terms of healthcare, there is almost no healthcare coverage in the remote rural areas of the region except Community-based Health Planning and Services (CHPS) zones, a primary healthcare facility dedicated to providing basic curative care, and usually manned by a trained community health nurse at best, or a community volunteer, at worst. Chronic shortages of equipment, medical supplies, and healthcare staff often worsen the situation. Currently, statistics suggest that there is one doctor for every 11,649 patient population and one nurse to 1,172 patient population in the region [22]. This situation is made worse by the refusal of Ghana’s highly trained medical doctors and nurses to accept posting to the region [22]. Like most parts of Ghana, the region is one of the areas where maternal mortality is still a worrying concern. The maternal mortality ratio as at 2013 stood at 161 deaths per 100,000 live births [22]. This is far higher than many regions in the southern part of Ghana. Only 55 % of births are attended by skilled birth attendants despite the fact that skilled maternal healthcare services, since 2003, have supposedly been provided free-of-charge at the point of delivery in health facilities [22].

In the UWR as in most parts of Ghana, gender-based role differentiation and male domination are entrenched and pervasive [31]. Men are in charge of decision-making and are seen as the breadwinners and women have less decision-making power both at the domestic sphere and the public realm [31]. It is within this context that this paper aims to examine the involvement of men in maternal healthcare.

### Participants

The main research participants were adult men. To gain multiple views on the involvement of men in maternal healthcare, however, the wives/partners of some of the men who participated in the study were interviewed. Also other key informants such as community leaders (chiefs, women's leaders, and assembly members), community health nurses, community health officers (CHOs), and mother-to-mother support group leaders, were also interviewed. In general, the ages of the participants, including those interviewed in key informants, ranged from 20 to 50 years. Majority of the participants had no formal education. Majority of the participants were also smallholder farmers with few others being professional teachers and community health nurses. All the participants, except some of the community health nurses, were married and had at least one child at the time of this study.

### Selection of participants

The seven study communities (four in Wa West and three in Lambussie-Karni districts) were purposively selected to capture a diversity of social and health situations that were largely representative of the region. In each of the study district, one urban community (i.e. community with a population of 5000 people or above), one rural community with at least one health facility, and one rural community without any health facility were selected. This was done after lists of all urban communities with a health facility, all rural communities with a health facility, and all rural communities without a health facility, were compiled in each district. From each of these categories, we purposively selected the community with the highest population.

A mix of purposive and convenient sampling techniques was however used to select individual research participants. Initially, we applied purposive sampling to select men whose spouses were either pregnant or lactating at the time of the research. Later on in the selection process, however, other men who expressed a desire to participate in the study were conveniently selected. The entire selection process was however based on a number of pre-set inclusion criteria: ease of recruitment, participant’s availability, participant’s willingness to participate in the study, and ability/capacity of a participant to consent to participate in the research.

The actual recruitment process involved advertising the study at vantage points (local community markets, churches and mosques) via community and religious leaders and CHOs, in addition to public announcement using the local community *gong gong* beaters (i.e. local community announcers). We acknowledge that the way in which the study was advertised could have introduced some bias in the selection of participants. For example, men who did not go to church or mosque were more likely not to hear about the study. However, we believe the diverse media we used to advertise the study such as beating the *gong gong* ensured that the majority of potential research participants heard about the study. Besides, the invitation was extended to all adult men and their spouses who met the essential inclusion criteria. The CHOs then helped the researchers to recruit interested individual participants for interviewing. Having grown up in the study communities, the CHOs were very conversant with the local cultural nuances and were therefore in a good position to advice the researchers on the selection and recruitment of participants, and arrange interview meetings.

### Data collection

Focus group discussions (FGDs) were the main data collection methods. This data collection technique was adopted partly because of its practical relevance in helping to reproduce men and women’s opinions on men’s involvement in issues of maternal healthcare in a normal peer-group interpersonal exchange [2, 3]. Other researchers have suggested that group setting often works well for generating talk about health, and that FGDs provide broader views about health and illness meanings [32]. In fact because FGDs were interactive, participants were able to query and challenge each other as well as explain themselves; hence offering validated data on the extent of consensus or diversity [33].

In all, 12 FGDs were completed with men. Groups were segmented by age (i.e. 20–30 years, and 31–50 years). We did this because initial discussions with CHOs suggested that there were age hierarchy conflicts in the study communities. In other words, younger men (20-30years) were unlikely to freely express their views in the presence of older men (31-50years) because of cultural norms, which require young people to listen to their elders. Segmenting discussants by similar age groups therefore ensured that each participant was comfortable expressing their opinions on all the issues as well as sharing their experiences within the group context with minimal hindrance. Groups consisted of 7–12 participants. Discussions in the FGDs lasted 1.30 to 2 hours, and ended when a point of saturation was reached i.e. when no new issues seemed to arise. All FGDs were held in the study communities. FGDs were held at locations convenient to both the participants and the researchers, and all the discussions were held in the local dialect – *Dagaari*. This was done because the literacy [written or spoken English] rates are low among the study participants. The researchers were all conversant and fluent in Dagaari and English. In the view of Grewal and Ritchie, a shared dialect could facilitate communication between the researched and the researcher [27]. The researchers’ ability to conduct interviews using a shared language with informants fostered rapport and social conversation with participants. The shared dialect also facilitated and smoothened the communication between the researchers and participants, as there was no need for a translator.

To complement the FGDs, in-depth interviews were also conducted with some of the men who participated in the FGDs and their spouses. These in-depth interviews were follow-up interviews. This was necessary because these men declined to discuss certain issues they considered personal and private in the group context. Instead, they expressed a desire to speak to the researchers alone. The selection of men for these individual in-depth interviews was therefore purposive, and was based on each participant expressing a desire to speak with the researchers alone. Indeed, in the literature, there are arguments that people may not necessarily tell the truth in any objective sense when it comes to sensitive issues such as health within a group context [34]. For this reason, the FGDs data were triangulated with individual interviews. A major advantage of this method was that it addressed sensitive issues such as personal experiences of childbirth and barriers to men’s involvement in maternity care. In total, 50 in-depth interviews - 25 with men and 25 with their spouses - were completed. We interviewed these women because we wanted to triangulate the information men gave in relation to their involvement in issues of maternal healthcare. Each interview lasted 30 to 40 min. Interviews were conducted in both Dagaari and English.

Lastly, key informant interviews (KIIs) were conducted with six chiefs, five women leaders, six assemblymen, five community health nurses, six community health officers, and two mother-to-mother support group leaders. Thus a total of 30 KIIs were completed. Generally, these KIIs sought to explore the views of other relevant stakeholders on the topic of male involvement in maternal healthcare. In this sense, the aims of the KIIs were not totally different from the other study participants. Rather, these KIIs complemented the FGDs and in-depth interviews with men and their spouses. In many instances, the findings from these KIIs gave further detail and nuance to the views and experiences of men and women. Interviews were conducted in both Dagaari and English, and lasted between 20 to 30 min.

#### Research instruments

Three open-ended thematic topic guides were designed and used. The first instrument was used in the FGDs with men. This instrument focused on exploring men’s understanding of the importance of skilled birth attendance during pregnancy and labour, men’s involvement in maternity care, and the barriers to, and enablers of, men’s involvement in maternal healthcare. The second instrument was used for in-depth interviews with women. This instrument focused on women's own perceptions about, and experiences with, men’s involvement in maternal healthcare. The last instrument was used to conduct interviews with key informants. This instrument focused on exploring the views of key informants on the topic of male involvement in maternal healthcare, and the challenges of, and opportunities for, involving men in maternal healthcare. All the instruments however had built-in flexibility that allowed for any pertinent but unexpected issues that arose during the discussion or interview process to be further probed. To ensure reliability, the instruments were pre-tested in one of the study communities. This helped to reframe questions, clarify and use more appropriate or easily understandable concepts. As the actual data collection progressed, the researchers also engaged in a continuous review of the questions and interview process to make sure that the questions were appropriate and rightly asked, and that the questions were understandable to research participants. All discussions and interviews were tape-recorded alongside hand-written field notes with the verbal consent of participants.

#### Data analysis

Qualitative data were analysed using Attride-Stirling’s thematic network analysis framework [35]. The Attride-Stirling thematic network analysis framework is a method for conducting thematic analysis of qualitative or textual data, which allows for open and methodical discovery of emergent concepts, themes and relationships through the application of principles of inductive reasoning to generating themes while also employing predetermined (deductive) code types to guide data analysis and interpretation [35]. This involved several steps.

Following the completion of interviews, all tape-recorded interviews were transcribed and all non-English transcripts translated into English. All transcripts and interview notes were then read and reviewed for overall understanding. This first step was completed with separate summaries for each transcript outlining the key points participants made. All transcripts were then exported into NVivo 9 qualitative data analysis software, where the data was both deductively and inductively coded. Data coding continued until theoretical saturation was reached (i.e. when no new concepts emerged from successive coding of data). The completed code structure was applied to develop and report themes. Themes simply represented some level of patterned response or meaning within the data set [36]. To ensure that the themes reflected the data, the data segments related to each theme were thoroughly examined. Where necessary, refinements were made. Where appropriate, verbatim quotations from interview transcripts were used to illustrate relevant themes.

#### Ethical considerations

Ethical approval for the study was obtained from the Ghana Health Service Ethical Review Committee (Protocol ID NO: GHS-ERC 18/11/13). In addition, informed written and verbal consents were obtained from all research participants. Individual participants were requested to sign or tomb print a written informed consent form. Participants (and there were only nine) who did not feel comfortable signing or tomb printing the written consent form were permitted to give verbal consent. Each verbal consent was witnessed by at least one family member or friend. To protect the identities and anonymity of participants, only pseudonyms have been used in the analysis and presentation of data.

## Results

The research participants’ views on men’s involvement in maternal healthcare, and the challenges of, and opportunities for, involving men converged on the following themes.

### Men’s perceptions of the importance of skilled maternal healthcare

Several of the men interviewed acknowledged the importance of women accessing and using skilled maternal healthcare services, especially ANC and skilled delivery. However, younger men and men in urban communities, perhaps because their levels of formal education were relatively higher than their rural and older counterparts, demonstrated greater awareness about the significance of women accessing and using skilled birthing services.

*I think it is important for every woman to go to hospital for check-up when they are pregnant. That way, the doctors can examine the pregnancy to make sure that everything is fine with the woman and her unborn child (Young Man, FGD).*

Nearly all women, in both rural and urban areas, also acknowledged the need for skilled care during pregnancy and at childbirth.

*I believe it is very important for every pregnant woman to seek proper care…I mean it is good to go to the doctor to check whether everything is fine with their pregnancy (Female Participant, IDI).*

Despite men’s awareness of the importance of women seeking skilled care during pregnancy or delivery, most of them reported that they do not usually accompany their wives to seek care neither do they encourage their wives to seek care, especially ANC during the first trimester of pregnancy and delivery in a health facility. Less than a quarter of male participants reported ever accompanying their wives for antenatal care or postnatal care check-up in a health facility. Most of the men who reported ever accompanying their wives to seek skilled maternal healthcare were, either younger (20-30years), educated or resident in urban towns. Focus discussions with men and in-depth interviews with women and community health nurses revealed several factors that limited men’s active involvement in maternity care.

### Men's involvement in maternal healthcare

Information was collected through FGDs, in-depth and key informant interviews about several aspects of men's involvement in maternal healthcare. In particular, men were asked whether their wives had any ANC checkups during the last pregnancy, whether they accompanied their partners to these checkups and the reasons why they did or did not accompany their wives to any ANC checkups and other maternal healthcare services. The results are presented in the next two sub-sections.

#### Barriers to men’s involvement

Most participants in the study reported four main barriers hindering men's involvement in maternal healthcare. These barriers include 1) masculinity and male role conflicts; 2) Cultural beliefs and practices; 3) health services factors such as unfavourable opening hours of services, poor attitudes of healthcare providers and lack of space to accommodate male partners in health facilities; and 4) high cost associated with accompanying women to seek maternity care.

#### Masculinity and male role conflicts

A major barrier to men’s involvement in maternal healthcare is the conflict between traditional definitions of men’s roles as breadwinners and their involvement in maternity care. In both FGDs and IDIs with men and women, it was reported that traditionally, men are seen as heads of households and breadwinners hence men’s focus is largely on economic activities. Maternal healthcare is usually seen as a 'feminine' domain and thus the responsibility of women’s.

*For me, I think it is the responsibility of my wife to go for ANC as I get busy with my farming activities. You know as a man, I am expected to provide food for my family…so if I spend time accompanying my wife to seek ANC, how would I be able to provide my family needs? Society and even my wife will mock me (Male participant, FGD).*

Men's role as family providers not only conflicts with modern demands for them to be involved in maternity care, but also acts as a barrier to accompanying their wives for maternal healthcare. Some men reported they were too busy for such tasks as accompanying their wives to seek ANC or PNC. In this regard, most men especially from rural communities felt it was time wasting to accompany their wives to seek care during pregnancy. This was often exacerbated by the fact that majority of the study's participants are predominantly smallholder farmers, and therefore too many demands on men's time especially during the rainy season compel them [men] to forgo accompanying their wives for maternal healthcare. Interviews with women largely corroborated the relatively low involvement of men in maternal healthcare.

*In this community, which man will stop his work and accompany you to go and check your pregnancy? My husband has never done it and most men don’t involve themselves with matters of pregnancy care…they say it is women’s affair (Female Participant, IDI).*

Some however said that they only got involved in situations where there are clinical complications such as protracted labour or delivery by caesarean section. Interviews with community health nurses suggested that men in urban areas were more willing to accompany or encourage their wives to seek skilled care than their rural counterparts. This was partly attributed to the fact that many urban men had relatively higher educational levels than their rural counterparts, and were therefore likely to be more aware of the importance of skilled care than rural men. Interviews with some urban men also suggested that they accompanied their wives to seek care because the health facility was not too distant. This notwithstanding, there was still a general consensus that most men are dissuaded from active participation in maternity care because of perceptions that caring for pregnancy and children is the preserve of women.

#### Cultural beliefs and practices

Apart from the fact that men felt their roles conflicted with the demands to be involved in maternal healthcare, other cultural factors inhibited men’s effective participation in maternity care. In several instances cultural standards were identified as barriers to men’s involvement. Several participants reported negative perceptions towards men attending ANC, DC and PNC services. For instance, men who accompanied their wives to ANC services were perceived as being dominated by their wives.

*The reason why some men do not involve themselves much in pregnancy care is because they do not want their peers to say that their wives are controlling them. You know in this community, there is the belief that if a man follows his wife to places like ANC then he is under the control of his wife (Female Participant, IDI).*

Also, women and healthcare providers reported that often men view ANCs and other maternity care services as designed and reserved for women. Men are thus embarrassed to find themselves in such “female” places. Interestingly, some of the women reported that they do not like to be seen with their male partner attending ANC.

*Personally, I would not like my husband to accompany me to seek care…normally when you go with your husband people in the community say you have used witchcraft to control your husband. The other thing is that when you go to the hospital with your husband, you cannot talk a lot with other women…you know men, they are always in a hurry. So I prefer to go alone in the company of other women (Female Participant, IDI).*

It was also reported in both FGDs and IDIs that some men do not encourage their women to seek skilled care due to various other reasons such as adherence to cultural and superstitious beliefs. Specifically, it was reported that when one discloses being pregnant within the first trimester, there is the possibility of losing the pregnancy through witchcraft. Both men and women also reported that attending ANC within the first trimester traditionally shows that the pregnant woman in question is not physically strong. One woman said:

*When I was pregnant for the first time and decided to attend ANC for the first trimester, my husband insulted me very well and told me that I was a lazy woman and that I could not resist the least pain. My mother-in-law also told me that these days ladies are just lazy because during her days, women could carry a whole nine-month pregnancy and would deliver successfully without attending ANC or seeing a doctor (Female Participant, IDI).*

The discussions here suggested that men sometimes draw on local cultural beliefs and practices to justify not only why they [men] should not be involved in maternal healthcare but also why their wives or partners should not seek skilled care during pregnancy or delivery.

#### Health services factors

Another key barrier to men’s involvement in maternal healthcare stemmed from alleged negative attitudes of health workers to men’s participation in maternity care, which to some extent appear to reinforce the marginalisation of men and the notion that pregnancy care is a female domain. Many participants reported that harsh and critical language directed at women and their husbands from skilled health professionals was a barrier to male participation. Harsh treatment of women by health providers was reported to discourage men from returning or accompanying their wives to seek care.

*I think one reason why men in this community do not involve themselves much in maternal healthcare is the attitude of the healthcare providers…I mean the nurses. I say this because I had a very bad experience three years ago when I went with my wife to see the midwife. The nurses were asking me what I wanted in the maternity ward…some even shouted at me to keep quiet when I wanted to tell the midwife how my wife behaved the night before our visit. Since that experience, I have told myself that I will not accompany my wife again to see the midwife (Male participant, FGD).*

Interviews with healthcare providers found that explanations for provider harshness and lack of respectful care to both women and men are often related to work overload resulting from chronic shortage of healthcare providers, lack of a functioning health infrastructure, poor supervision, and poor working conditions including lack of motivation and low salaries of care providers.

Also long waiting time was reported as a major barrier to men’s involvement. Frequently women have to wait for a long time before receiving care because of burdensome administrative procedures and a lack of appointment system, which result in poor patient/client care in healthcare facilities. Men, who frequently are in the paid workforce, are often not in a position to spend virtually the entire day waiting for their wives to receive care. As a result, some male participants reported that they would readily accompany their wives for maternity care if they would be given priority in the queue before women who were unaccompanied by their husbands.

Male participants also argued that a major obstacle to their involvement in maternal healthcare stems from the maternal healthcare services delivery system itself, which is almost exclusively oriented to women and often provide little or no information about male involvement in maternal healthcare. The lack of space to accommodate male partners in clinics was further reported to adversely impact male involvement. Clinics are often unable to concurrently accommodate pregnant women and their partners because of a lack of space, and gender specific services to address uniquely male issues do not exist. Husbands who accompany their wives for such care are often given poor reception, made to stand or sit outside the maternity ward and worse still, are usually not given explanations on the treatment their wives receive. One male participant narrated his experience thus:

*One day, I followed my wife to a health facility. Upon entering the labour ward, the nurse ordered me to go out in a way as if I was not a human being. She continually shouted at me and pointed at the door for me to go and wait outside. After this experience, I have never escorted my wife again for maternal care (IDI).*

Another participant reported:

*Sometimes, the nurses are always so harsh and they don’t want to see a man (husband) inside the maternity ward. There is nothing you can do because you cannot see her (wife) although you wish to be near her, so it is better for you the husband to stay away (at home) and do some other things that can help her after delivery (Male Participant, FGD).*

Consequently, it was reported that most men choose to stay away from accompanying their spouses to seek maternity care, feeling that they are not needed at the health facilities where their women go to receive care.

#### High costs

Several participants also reported the high cost associated with seeking skilled maternal healthcare services as a disincentive to men’s involvement in maternity care. According to this account, although maternal healthcare is supposedly free at the point of delivery, clients often have to pay illegal fees to the staff in order to receive care. Also the cost involved in arranging appropriate transportation to travel to receive the free services was reported to be very high. This is often compounded by poor road network and the lack of appropriate transportation particularly in many rural areas. Men who had no means of transport and who did not want to go through this stress of travelling long distances on foot to seek maternal healthcare often resort to the use of local options such as unskilled traditional birth attendants in their community. One male participant narrated his experience:

*We live in a place where transport is hard to come by. When your wife is in labour and you need to send her to the health facility, you find it difficult to get one. When you get a motorcycle, the owner will request that you buy fuel which some of us cannot get. The worse thing is that the motorbikes and tricycles are not common here; only the rich have them (Male Participant, FGD).*

Thus the costs of transportation to and from health facilities and the opportunity cost of time can be very high enough to deter men from involving in issues of maternal healthcare. It was however acknowledged by most participants that the issue of high transport costs and difficulties with getting appropriate transportation is largely a rural problem. This is due to the fact that large proportions of the rural population live in poverty and have difficulty paying for such services. But even in urban settings where public and private transport services are mostly available, it was reported that urban public transport presents a number of challenges. For instance, it was reported that sometimes a pregnant woman still has to walk or be hand-carried from home to bus terminus or from bus terminus to the healthcare facility when public transport is used. Also, delays associated with the operations of these transport services often cause further delays in reaching a health facility. It was also reported that expectant mothers often endure traffic jams on the way, and this results in delays in getting to healthcare centres.

#### Facilitators of men’s involvement

Despite the low level of men’s involvement in maternal healthcare in the study communities and the challenges to men’s involvement, few of the men thought they had a role to play when their wives are pregnant. Indeed, some men reported defying gender stereotypes to accompanying their wives to seek care and to providing support and encouragement to their wives during and after pregnancy and childbirth.

*Me I was with my wife during all her ANC visits and even during her delivery. My friends made fun of it but you know I did not mind them. My involvement gave my wife support and encouragement so that when she was pushing the child, she pushed it with happiness and comfort since she knew her husband was by her (Male Participant, IDI).*

One male participant also said:

*Me…I followed my wife to the health centre where she was going to deliver. After all, she is carrying my pregnancy and the expectant child belongs to both of us. Truly, I cannot share the pain of pregnancy but I have to support my wife during pregnancy, childbirth and even in child rearing and caring. It is the responsibility of both of us (Male Participant, IDI).*

In FGDs, IDIs and KIIs, participants identified a number of factors that could facilitate men’s involvement in maternity care. These include 1) community mobilisation and engagement to promote greater male involvement in maternity care; 2) promotion of respectful and patient-centred care; and 3) health education.

#### Community mobilisation and engagement to promote male involvement

In both focus groups and interviews, it was reported that maternal health issues in many communities were still largely treated as women’s business, and maternity wards as spaces exclusively meant for women. Consequently, there has been very little focus on men and their involvement in helping women access care. However, several men and women reported that not only do husbands influence women’s healthcare-seeking decisions through for example financing, but also they exercise considerable power in either permitting or restricting women’s access to, and use of services. Several participants therefore called for community mobilisation and engagement initiatives and programmes that promote men’s involvement in maternal healthcare.

*My view is that if the healthcare providers want every woman in this community to attend antenatal clinic or deliver their babies at the hospital, then they need to talk to we the men too and involve us more to understand why it is important. I say this because we the men are often responsible for the pregnancy and therefore we have a lot of say in terms of how the pregnancy is cared for or how the baby should be born.* (Male Participant, FGD).

Another participant said:

*You know many of us men still think that it is only the duty of the women to care for the pregnancy. Some of us also do not still understand the importance of skilled attendance at birth… and you know what we men do not know can actually hurt women’s health. So the health people must engage we the men more* (Male Participant, FGD).

Many of the healthcare providers and community leaders interviewed in this study agreed that programmes directed towards improving women’s access and use of skilled maternity care services must involve men. For these participants, many communities in Ghana are still characterised by patriarchy and machismo such that men, especially husbands, are usually the most influential household decision-makers, including regulating women’s mobility and autonomy in accessing and using skilled care services.

*I believe one problem is the failure of the healthcare system to actively engage men on issues of maternal health. But you see, we still live in a country where men have more control over household decisions, including decisions like how many children to have and whether a woman should give birth in the hospital. So I think that if we want to ensure that all women have access to or use skilled maternal healthcare services, then we the healthcare providers must also engage the men in the process* (Female Healthcare Provider, KII).

Where there has been involvement, it was reported that men’s involvement is often defined to only mean accompanying or paying for care of pregnant women. However, these measures were said to provide little information about how else a husband may be involved in his wife’s pregnancy experience, and how engaged a husband may or may not be when he accompanies his wife for care. The accounts of several women and key informants in the study suggested that actively engaging men on issues of maternal health will make men feel a sense of partnership and collective ownership of the maternal health services that are being offered at health facilities, and address issues of socio-cultural barriers and resolve conflicts between men and healthcare providers.

#### Promoting respectful and patient-centred care

Poor relationships between healthcare providers and women and their spouses is one reason why some men are reluctant to accompany their wives to seek maternity care. To reverse this and to involve men in maternity care, many participants proposed the promotion of a regime of care that is respectful and patient-centred.

*One problem is that the health facilities and the workers are supposed to serve our needs. Unfortunately, you will go to the hospital and the workers will treat you very badly. I think if they really want men to be involved, then they the healthcare providers have to change the way they treat us…they should be more friendly, compassionate and treat us well* (Male Participant, FGD).

Another participant said:

*For us men to be more involved, I think the health workers need to learn how to treat us when we visit the clinic. I say this because of my past experience …at one of the health centres there used to be one midwife…she was very nice to all the men and women who visited the health centre …she talked nicely to people especially men. Even when you make a mistake, she will not shout at you; she will take her time to talk to you. So a time came that all the pregnant women wanted only that midwife to attend to them. Men were also very willing to take their wives to see that midwife. But now, she has been sent to a different place, and most of the new people are not friendly at all. That is why many men are not accompanying their wives to seek care* (Male Participant, IDI).

Many of the healthcare providers interviewed also suggested that improving the doctor-patient relationship at healthcare facilities is an important approach to involving men in maternal healthcare.

*To get many men involved, I think we the healthcare providers need to focus on how we treat the men and women who come to us for help. We must focus on making all our facilities and especially labour wards patient-friendly* (Male Healthcare Provider, KII).

One mother-to-mother support group leader also said:

*I think the healthcare providers need to focus on improving the relationship between caregivers and women and their spouses. They need to provide more patient-centred care that is able to address the needs of women, accord the men who accompany them to the health facilities respect and assure dignity anytime they visit a health facility* (Female Participant, KII).

#### Health education

Several participants also proposed more health education as a strategy to encourage men to be more involved in maternal healthcare. According to this account, involving more men on issues of maternal healthcare must be preceded and/ or accompanied by a very aggressive education of men.

*There is still ignorance on the part of many men about health issues including maternal health. I believe the way to make progress is to increase health education campaigns using the radio and community durbars to educate men about the need for them to encourage their wives to seek proper care during pregnancy* (Male Participant, IDI).

One male participant also said:

*The problem is that there are many men…especially in rural areas who have no formal education. Such men do not always understand the risks involved in getting pregnant and giving birth…they also have funny cultural beliefs about hospital births. So if the health people want we the men to be actively involved then they must intensify their health education campaigns to educate men to understand the importance of their involvement in seeking care during pregnancy or childbirth and to do away with traditional and cultural beliefs that prevent them from actively taking part in issues of maternity care* (Male Participant, FGD).

## Discussion

This qualitative research paper explored the barriers to, and opportunities for men's involvement in maternal healthcare in the Upper West Region of Ghana. Findings suggest that although men recognised the importance of skilled care during pregnancy and childbirth, many did not actively involve themselves in issues of maternal healthcare unless complications set in during pregnancy or labour. This is consistent with previous findings from Kenya, which suggested that although men recognised the benefits of their involvement in maternity care, few did in practice involve themselves in accompanying, encouraging and supporting their wives to seek care [1].

Four main barriers to men’s involvement were identified. These included perceptions that pregnancy care is a female role while men are family providers, negative cultural beliefs and practices both in relation to skilled maternity care and men’s involvement, health services factors such as unfavourable opening hours of services, poor attitudes of healthcare providers and lack of space to accommodate male partners in health facilities, and the high cost associated with accompanying women to seek maternity care. Previous studies have documented similar barriers in other parts of Africa [1, 19, 20]. Despite these barriers, few men reported their active involvement in maternity care and suggested strategies by which men could be motivated and encouraged to be more involved. These strategies included community mobilisation initiatives to promote greater male involvement in maternity care, respectful and patient-centred care, and health education.

Together, the findings in this paper suggest that although there are challenges to men’s active involvement in promoting access to skilled care, there are equally opportunities for instituting the necessary programmatic and policy initiatives to get many men involved in the process. For instance, several explanations for providers’ harshness and disrespectful care to patients were offered by healthcare providers, including providers’ low salaries, lack of a functioning health infrastructure and a critical shortage of healthcare providers. While these are certainly realities working in sub-Saharan Africa [20], they suggest the urgent need for further training in nursing, midwifery and medical schools on the principles of patient and family-centred care, combined with improved customer care communications. As some previous studies have shown, a focus on training of health personnel on ‘public relations’ could built trust and restore confidence in the healthcare system [37, 38]. Of course, training and education alone might be insufficient to address this challenge. Therefore, there will be the need for effective and supportive leadership, which shows the way forward in terms of decent behaviour towards the clients. In this regard, we recommend the strengthening and/ or institutionalisation of an effective reward and sanctions regime whereby caregivers whose practices promote women’s access are rewarded, while those who contravene good practice and ethical standard of care, thereby obstructing women’s healthcare seeking choices and men’s involvement, are penalised. This recommendation further suggests the need for training and education in healthcare ethics and in communication skills for health professionals.

Limited physical space to accommodate male partners is one of the reasons why healthcare providers have difficulties incorporating male partners in maternity care. This situation is worsened when healthcare workers are understaffed, underpaid and overworked. This suggests the need to increase the number of trained healthcare providers and to improve the availability of physical infrastructure. In particular existing and future designs of maternity wards and health facilities could be made more male and couple-friendly. For example, separate spaces could be created for only couples. Similarly, separate waiting areas could be created for men alone when they accompany their wives to access maternal healthcare. This could reduce the discomfort some men feel when they accompany their spouses to seek maternal healthcare and are required to sit among women or other patients waiting to receive care. Also, there will be the need for better planning and the institutionalisation of a booking or appointment regime – something currently absent in most health facilities in Ghana - so as to reduce overcrowding in health facilities and long waiting times before care is received. This of course could be problematic to implement in the short term especially in remote areas where communication and transportation services might be lacking [39]. However, in the long term, healthcare facilities can establish systems in local communities that could be used to facilitate booking before attendance. For instance, community health workers could be trained and equipped with mobile communication devices. These community health workers could then be tasked with the responsibility of receiving booking requests from women in the community and then communicating such requests to the appropriate healthcare facilities.

Also, in the context of the study communities where men can prevent women from accessing maternal healthcare, and where there is convergence of opinion on the need to involve men as partners in maternal health, the health service in Ghana could initiate programmes to engage men on maternal health issues. Initiatives for involving men do not need to have a primary focus on increasing men’s perceptions of their responsibility in their wives’ maternity care. Some husbands are conscious of the need for men to participate in their wives’ maternity care. The challenge lies in bridging the gap between awareness and responsibility on one hand and participation on the other. This brings to the fore a programmatic and policy question: how should men’s participation be defined and what kind of participation should be encouraged in achieving better maternal health outcomes? It is essential to define men and husbands’ involvement in maternal healthcare more broadly than has often been the case. The definition has to be contextually valid and should recognise the role of men within the cultural constraints they live and the resources available to them, while at the same time taking into account the desires and needs of women. Initiatives for involving men could include couple counselling, dialogue with men, and health education. Health education campaigns must not only communicate the importance of women delivering their babies with a relatively well-resourced skilled health professional in attendance, and the benefits of men’s involvement in maternal healthcare. Such campaigns must also challenge negative socio-cultural beliefs and practices that both frame maternity care as the business of women, and constrain men’s ability to play more supportive roles in maternal healthcare. In this regard, traditional and religious leaders such as local community chiefs, Imams and pastors – who usually wield considerable power and influence at the community level - could be involved to mobilise communities and lead education campaigns that seek to encourage more men to be involved in promoting women’s access to and use of skilled birthing services. While, cultural perceptions about the role of men as breadwinners and maternity care as a feminine domain could potentially hinder men’s active involvement [2, 3, 33], the findings from this study suggest that some men are actually willing to be involved if only some of the other barriers they face are addressed.

Finally, although the government of Ghana has tried to eliminate user-fees for maternal healthcare services at the point of delivery, high costs and difficulties with arranging appropriate transportation to transport pregnant women to health facilities is one reason why many rural men do not actively involve themselves in maternal healthcare. Under the current regime of maternity care in Ghana, ambulatory services are not covered by the fee-exemption policy. Ambulatory services are also very limited, often ineffective, and usually restricted to urban centres. In rural communities where there are often no healthcare facilities, and where appropriate transportation is difficult and expensive to arrange in the event of obstetric emergency, the men, women and healthcare providers interviewed in this study reported that getting an appropriate transport to travel to seek care often presents a challenge. To address this challenge, the government or the health service could invest more resources into providing free or subsidised but efficient and responsive ambulatory services, particularly in remote communities. Providing subsidised and responsive ambulance services would reduce the cost involved in arranging appropriate transportation and thus make it possible for more births to take place in hospitals with the active involvement of both women and men.

The findings and recommendations in this paper should however be read against the backdrop of certain limitations. The research reported in this paper was conducted in a context where patriarchy and male domination is prevalent. The limitation of applying the findings to other contexts such as matrilineal or more egalitarian societies is therefore acknowledged. This notwithstanding, important lessons can be drawn from the findings in this paper to inform policies that seek to encourage men to be involved in promoting women’s access to and use of skilled maternity care services in other parts of the country and beyond.

## Conclusion

This study is one of the few studies in Ghana to have focused on exploring and understanding the barriers to, and opportunities for, involving men in maternal healthcare. Findings suggest that many men did recognise the importance of skilled care during pregnancy and childbirth and the benefits of their involvement in issues of maternity care. Except situations of obstetric complications, however, few men actually involved themselves in issues of maternal healthcare. The qualitative data suggest that men were often excluded from participating in routine check-ups partly because their societies consider maternal healthcare as women’s business, and partly because the medical system does not accommodate them. As efforts to improve access to and use of skilled maternal healthcare services continue, it is essential that the barriers to men’s involvement are addressed so that men who are often important decisions-makers within the household and family settings are involved to facilitate women’s access to maternal healthcare. Men’s involvement in issues of maternal healthcare could be important for increasing in them an understanding of the relevance of women’s access to, and use of, skilled birthing services in a timely manner. This can enable men to play more supportive roles in the area of maternal healthcare access.
